# Predation drives complex eco-evolutionary dynamics in sexually selected traits

**DOI:** 10.1371/journal.pbio.3002059

**Published:** 2023-04-03

**Authors:** Brian A. Lerch, Maria R. Servedio

**Affiliations:** Department of Biology, University of North Carolina at Chapel Hill, Chapel Hill, North Carolina, United States of America; University of California, Davis, UNITED STATES

## Abstract

Predation plays a role in preventing the evolution of ever more complicated sexual displays, because such displays often increase an individual’s predation risk. Sexual selection theory, however, omits a key feature of predation in modeling costs to sexually selected traits: Predation is density dependent. As a result of this density dependence, predator–prey dynamics should feed back into the evolution of sexual displays, which, in turn, feeds back into predator–prey dynamics. Here, we develop both population and quantitative genetic models of sexual selection that explicitly link the evolution of sexual displays with predator–prey dynamics. Our primary result is that predation can drive eco-evolutionary cycles in sexually selected traits. We also show that mechanistically modeling the cost to sexual displays as predation leads to novel outcomes such as the maintenance of polymorphism in sexual displays and alters ecological dynamics by muting prey cycles. These results suggest predation as a potential mechanism to maintain variation in sexual displays and underscore that short-term studies of sexual display evolution may not accurately predict long-run dynamics. Further, they demonstrate that a common verbal model (that predation limits sexual displays) with widespread empirical support can result in unappreciated, complex dynamics due to the density-dependent nature of predation.

## Abstract 2

It’s long been known that predation acts as a major constraint on the evolution of sexual displays, but the implications of interactions between predator-prey dynamics and sexual selection are less well explored; here, population and quantitative genetic models of sexual selection reveal that predation can drive eco-evolutionary cycles in sexually selected traits.

## Introduction

Widespread evidence suggests that sexual displays used to attract mates often increase an individual’s risk of being predated [1–11]. Such costs inhibit the evolution of sexual displays across various sensory modalities in taxa ranging from fish to frogs to insects [12–17]. Despite empirical evidence that predation serves as a major constraint on the evolution of sexual displays, theoretical models of sexual selection that mechanistically model predation as the source of natural selection have not been developed. Rather, past models treat costs of expressing sexual displays phenomenologically, assuming fixed population densities and frequency-independent selection against the display [18,19]. These assumptions are incompatible with predation being the cost to expressing displays because predation is density dependent, and thus, viability selection against sexual displays should also be density dependent. Density-dependent selection against sexual displays could explain the maintenance of genetic variation in display traits despite persistent positive sexual selection through female choice (the “lek paradox”) [20,21], especially if natural selection fluctuates in strength due to coupling with predator–prey cycles.

From an ecological perspective, important consequences of evolution in predator–prey systems are well known. For example, optimal foraging behavior may shape functional responses (how predation rate changes with prey density) [22–24] and coevolution helps structure predator–prey communities [25–28]. Sexual behavior can be important for ecological dynamics through interactions with predation [29,30] and by mediating population growth [31–35] and extinction risk [36–42].

Mechanistically modeling interactions between predation and sexual selection is of particular importance because evolution may occur on ecological timescales [43–47], with the resulting interplay between changes in population density and trait evolution driving eco-evolutionary feedbacks (wherein evolution alters population dynamics, which, in turn, alter evolution [48–52]). Sexually selected traits can evolve on ecological timescales [53–58], but few studies have considered the effects of eco-evolutionary dynamics on such traits [59,60]. In competitive systems, rapid evolution via sexual selection may facilitate coexistence [61]. In predator–prey systems, eco-evolutionary feedbacks have been demonstrated empirically [62–68], although there is no direct evidence for eco-evolutionary feedbacks affecting sexually selected traits in these systems. However, the strong influence of displays on predation risk (which, e.g., doubles in Trinidadian guppies [7]; *Poecilia reticulata*) suggests that sexual displays may also have a strong influence on predator populations. In general, prey evolution can either drive or dampen predator–prey cycles [69–73]. However, previous work tends to model predation risk trading off with growth [69,70,74] or competitive ability [71], with different trade-offs leading to qualitatively different outcomes [75–77]. The empirically justified mechanism of a sexually selected benefit to a display trait differs from previously explored trade-offs because there is not necessarily an ecological benefit to expressing a sexual display.

Here, we develop eco-evolutionary models of sexual selection that explicitly treat the cost to expressing a display as increasing predation risk. To best connect our results to both the evolutionary and ecological literature, we develop (1) a model that maximizes ecological realism and aligns with literature on eco-evolutionary feedbacks in predator–prey systems using a continuous-time, quantitative genetic framework and (2) a model that maximizes genetic realism and aligns with important contributions in the sexual selection literature using a discrete-time, population genetic framework. The essential features of both approaches are that they track prey density, predator density, and a sexual display that males may express to increase their attractiveness to females, but which puts them at higher risk of predation. We show that sexual selection qualitatively alters population dynamics and that predator–prey dynamics, in turn, generate novel evolutionary outcomes, demonstrating that rich eco-evolutionary dynamics can result from sexual selection.

## Results

**Fig 1:**
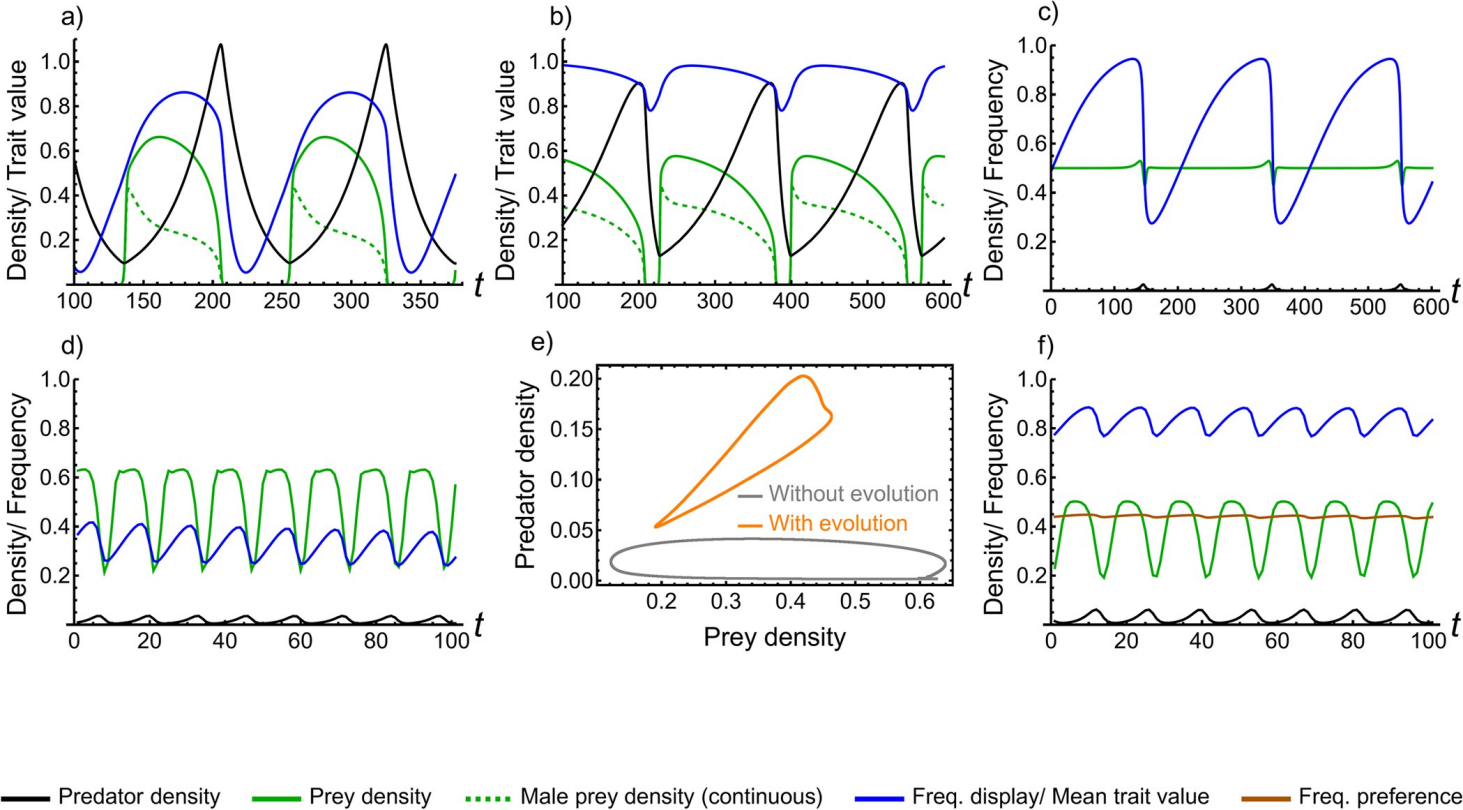
Eco-evolutionary cycles of predator–prey systems coupled with sexual selection. (**a**-**d**, **f**) show population density and trait frequency/value through time (see legend). (**a**) Continuous model with high amplitude eco-evolutionary cycles: prey growth rate *r*_*c*_ = 2, handling time *t*_*h*_ = 1, display-dependent predation cost *s*_*c*_ = 10, basal predation rate *b*_*c*_ = 5, preference strength *a*_*c*_ = 5, conversion efficiency *c*_*c*_ = 0.1, predator mortality rate *m* = 0.05, prey genetic variation *σ* = 0.1. (**b**) Continuous model with eco-evolutionary cycles showing long lag in the display cycle: *r*_*c*_ = 3, *t*_*h*_ = 0.75, *s*_*c*_ = 5, *b*_*c*_ = 8, *a*_*c*_ = 10, *c*_*c*_ = 0.1, *m* = 0.1, *σ* = 0.1. (**c**) Discrete model with high period eco-evolutionary cycles: *r*_*d*_ = 4, *s*_*d*_ = 15, *b*_*d*_ = 15, *a*_*d*_ = 1.05, *c*_*d*_ = 0.1. (**d**) Discrete model with low amplitude, low period, eco-evolutionary cycles: *r*_*d*_ = 5, *s*_*d*_ = 5, *b*_*d*_ = 20, *a*_*d*_ = 1.25, *c*_*d*_ = 0.1. (**e**) Comparison between predator–prey cycles with (orange) and without (gray) evolution (see Methods for model details) in the discrete model. Parameters same as (**d**). (**f**) Eco-evolutionary cycles in the Fisher process model: *r*_*d*_ = 4, *s*_*d*_ = 4, *b*_*d*_ = 10, *a*_*d*_ = 2, *c*_*d*_ = 0.1. This Figure can be generated using S1 Code.

Our primary result (regardless of model details; see Methods) is that predation can drive complex evolutionary dynamics in sexual displays (Fig 1). We see endogenous cycles previously undescribed in comparable models of sexual selection. When predator density is low, viability selection is relaxed so both prey density and display frequency increase. Once this occurs, predator density also increases, thus driving a decline in prey density and strengthening selection against the display trait, decreasing its frequency. This general pattern occurs in both the discrete and continuous model (Fig 1).

**Fig 2:**
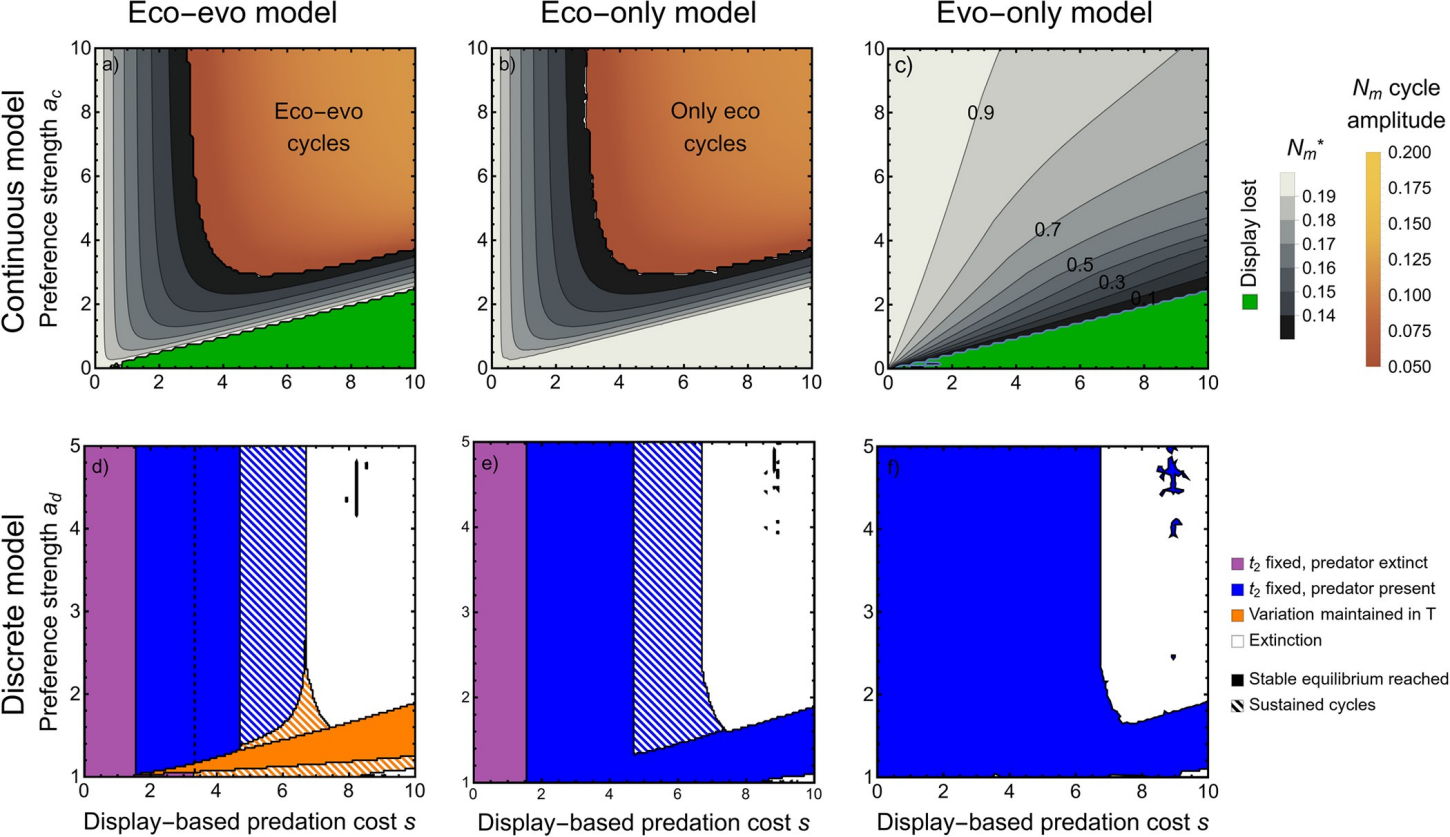
The role of eco-evolutionary dynamics as a function of preference strength and predation cost. Comparing to S1 Fig demonstrates the importance of eco-evolutionary dynamics in setting long-run density and trait values if one were to consider either model in isolation. (**a**–**c**) The continuous model with *r*_*c*_ = 2, *b*_*c*_ = 5, *σ* = 0.1, *c*_*c*_ = 0.1, *m* = 0.1, *t*_*h*_ = 0.5. (**a**) Equilibrium outcomes from the full, eco-evolutionary model. (**b**) Outcomes with only predator–prey dynamics. (**c**) Outcomes with only sexual selection. Green indicates that the sexual display is lost. Gray indicates that a stable equilibrium is reached. For (**a**, **b**), gray contours correspond to equilibrium male prey density *N*_m_* (see legend). For (**c**), gray contours correspond to the mean display trait value at equilibrium (compare to Fig 3). Yellow-orange indicates sustained cycles, with the shading giving the amplitude of the male prey’s cycle (see legend), which correlates with period and the amplitude cycles in all other variables (S2 Fig). Note that, although subtle, cycles in the purely ecological model are higher amplitude (lighter shading). (**d**–**f**) The discrete model with *r*_*d*_ = 5, *b*_*d*_ = 15, *c*_*d*_ = 0.1. (**d**) Equilibrium outcomes from the full, eco-evolutionary model. Purple indicates the sexual display fixes and predators go extinct. Blue indicates the sexual display fixes and predators persist. Orange indicates variation is maintained in the sexual display. White indicates extinction. Solid regions correspond to reaching a stable equilibrium, striped regions indicate sustained cycles. (**e**) Outcomes with only predator–prey dynamics (blue means the predator persists). (**f**) Outcomes with only display evolution (blue means the display fixes). That (**b**) is much more similar to (**a**) (and **e** to **d**) than is true in S1 Fig indicates that ecological interactions are altered in the full eco-evolutionary model due to the way that densities and trait values are set, not just due to eco-evolutionary feedbacks per se. In contrast, that (**c**) is comparably different from (**a**) as in S1 Fig indicates that eco-evolutionary feedbacks per se are also responsible for altering evolutionary outcomes. This Figure can be generated using S1 Code.

Feedbacks inherent to predator–prey dynamics are essential in the generation of evolutionary cycles in sexual displays. Without explicitly including population dynamics, evolutionary cycles are never observed (Fig 2, right column). Furthermore, stable polymorphism in the sexual display can occur in the discrete model; such polymorphism relies upon the density-dependent nature of predation and thus requires population dynamics (Fig 2D–2F). Clearly, ecology can have important influences on sexual selection, leading to qualitatively distinct outcomes. Evolving sexual displays also alter predator–prey dynamics. Overall, evolution tends to decrease the amplitude of prey cycles and the range over which they occur (Figs 1E and S1), since prey can evolve to decrease their predation risk in response to increased predation by muting their displays. It can be further shown that rather than eco-evolutionary feedbacks per se, the most important role of eco-evolutionary dynamics is to determine emergent long-run trait values/densities. That is, choosing fixed, arbitrary long-run values for predator and prey densities or for evolutionary traits, and considering only the evolutionary or ecological submodels, respectively, results in dramatically different qualitative dynamics than considering the full eco-evolutionary model (compare S1 Fig to Fig 2).

**Fig 3:**
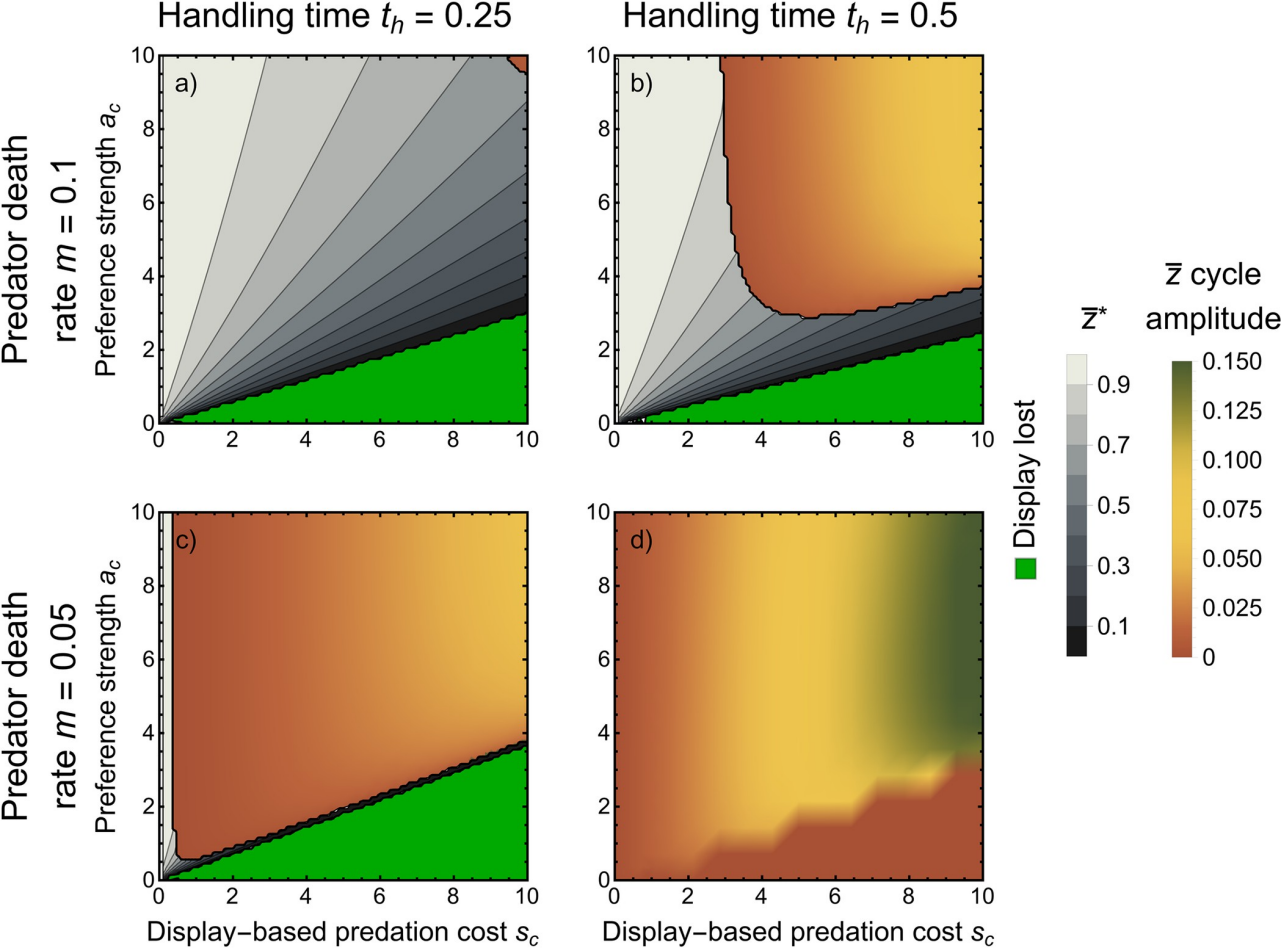
Eco-evolutionary outcomes from the continuous model. Horizontal axis is display-based predation cost *s*_*c*_, and vertical axis is preference strength *a*_*c*_. Each panel represents a different predator death rate *m* or handing time *t*_*h*_. Regions have the same meaning as Fig 2, except in grayscale regions, lighter colors correspond to higher display trait value at equilibrium and in dark green-yellow-orange regions dark green and yellow colors correspond to higher amplitude of cycles in the display trait (which correlates to period and the amplitude of cycles in other state variables; S2 Fig). Note that the trait is lost and cycles are purely ecological in the bottom right, rust-colored region of (**b**). *r*_*c*_ = 2, *b*_*c*_ = 5, *σ* = 0.1, *c*_*c*_ = 0.1. This Figure can be generated using S1 Code.

In the continuous model, fluctuations in the relative strengths of natural and sexual selection are enhanced by both display-based predation costs *s*_*c*_ and preference strengths *a*_*c*_ being large, thus making sustained eco-evolutionary cycles more likely (Fig 3). As expected with predator–prey cycles, faster saturation of predation with prey density (higher *t*_*h*_) makes predators most efficient when prey are rare (and least efficient when they are common), leading to more frequent and exaggerated cycles (Fig 3). Low predator death rate *m* means that predator density responds slowly to decreasing predation rate, also making cycles more likely and exaggerated (Fig 3). The amplitude of cycles is greatest with high display costs *s*_*c*_ but is relatively insensitive to preference strength (Fig 3). In ecological models, the lag from prey peak to predator peak is shorter than one-quarter of the period length [78]. With an evolving, sexually selected trait, however, we find the lag is longer (typically between one-quarter and one-half the period length; S2E Fig), but not as long as reversed cycles (predator-led) that occur in other evolving predator–prey systems [63,79,80]. When cycles do not occur in the continuous model, prey and predators often coexist at a stable equilibrium. In this case, increasing the cost of a display (*s*_*c*_) and decreasing the preference strength (*a*_*c*_) decrease the mean display trait value at equilibrium (gray, Fig 3). While increasing the basal predation rate *b*_*c*_ results in more of parameter space with eco-evolutionary cycles, prey growth rate *r*_*c*_ and genetic variation *σ* do not have a large effect on equilibrium outcomes (S3 Fig).

**Fig 4:**
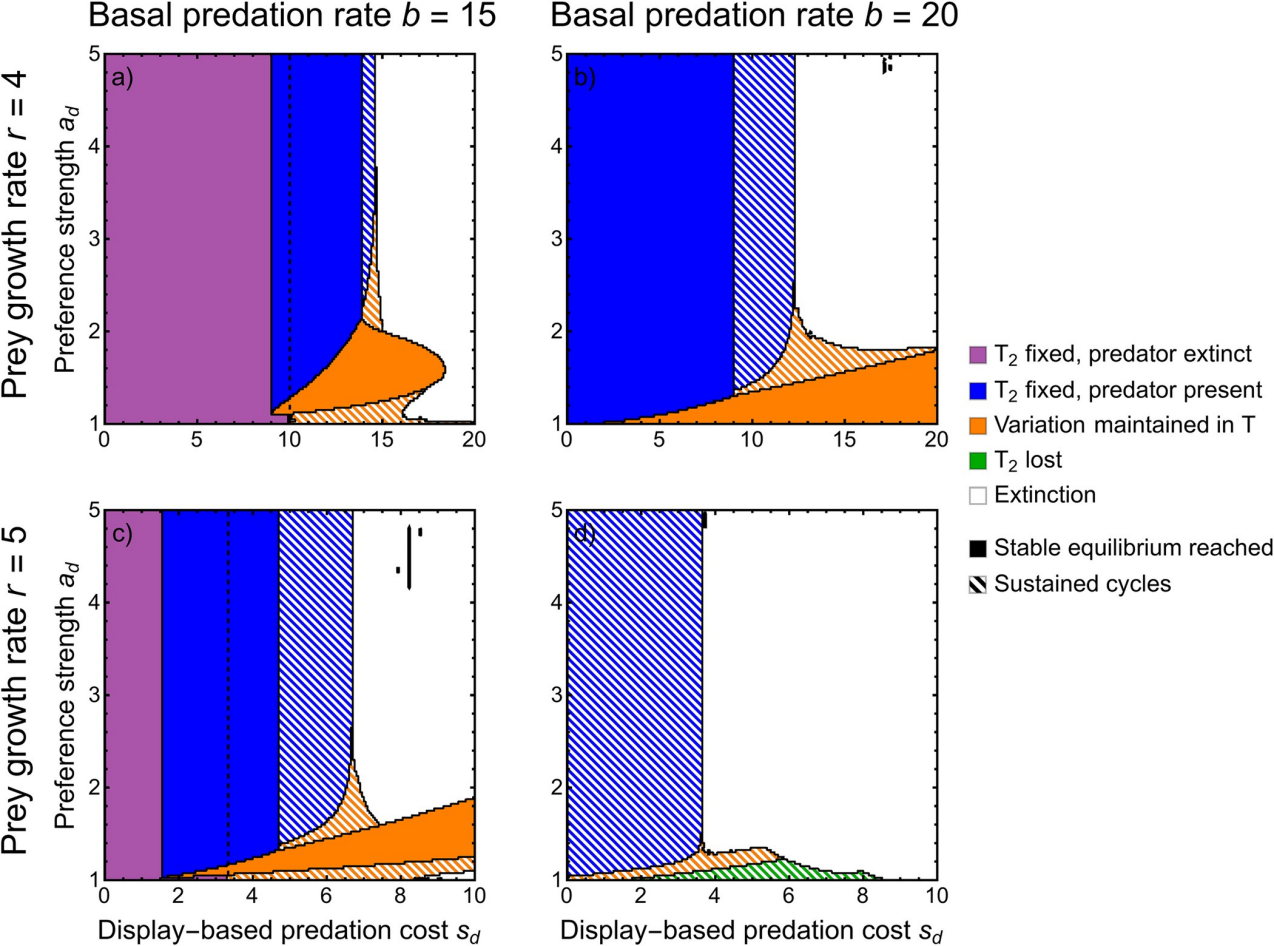
Eco-evolutionary outcomes from the discrete model. Horizontal axis is display-based predation cost *s*_*d*_, and vertical axis is preference strength *a*_*d*_. Each panel represents a different basal predation rate *b*_*d*_ and prey growth rate *r*_*d*_. Colors and stripes have the same meaning as in Fig 2. Striped blue and green indicates only ecological cycles, whereas striped orange corresponds to eco-evolutionary cycles. White regions indicate that density becomes negative (which we interpret as extinction). If the model did remain well behaved, we believe that this region would mostly consist of eco-evolutionary cycles; however, the cycle amplitude is too large and negative densities result. The dashed black vertical line seen in some panels is the display-based predation cost below, which predator extinction is stable (thus, regions of blue or orange to the left of this line indicate bistability). Note that the scale of the horizontal axis changes across rows. *c*_*d*_ = 0.1. This Figure can be generated using S1 Code.

Eco-evolutionary models of predator–prey systems typically lack detail regarding genetic architecture and covariance even though their dynamics depend critically on genetic parameters [73,75,81–83]. Our discrete model assesses the importance of explicit genetic detail and allows genetic variation to change naturally with time. In the discrete model, eco-evolutionary cycles neighbor a region where polymorphism is maintained in the sexual display (Fig 4; solid orange) and are only observed when the display-dependent predation cost *s*_*d*_ is much larger than the preference strength *a*_*d*_ (Fig 4; striped orange). This occurs because a high display-based predation cost *s*_*d*_ strongly couples high predator density to a decrease in display frequency. Again, we find that higher amplitude eco-evolutionary cycles occur when the cost of displays is stronger, though now low preference strength also leads to larger cycles (S4 Fig). Once preference strength becomes too strong or the predation cost too weak, the sexual display goes to fixation (purple and blue, Fig 4). As the display-based predation cost *s*_*d*_ increases, predation rate increases, causing transitions from predator extinction with prey sexual display fixed (when the basal predation rate alone is too low for predator persistence) to either a stable equilibrium of both predators and prey with the sexual display fixed or maintained variation in the display trait with eco-evolutionary cycles, depending on the preference strength *a*_*d*_. If the predation cost is too large, both species go extinct. Prey extinction at high predation cost *s*_*d*_ corresponds to evolutionary suicide: If not for the evolution of the sexual display, the prey would not suffer from the predation cost and thus could persist. Sexual selection has been discussed as a driver of evolutionary suicide [37], a result supported in the ostracod fossil record [41,42]; these results add predation as a mechanism by which female choice can drive populations extinct in models [84].

**Fig 5:**
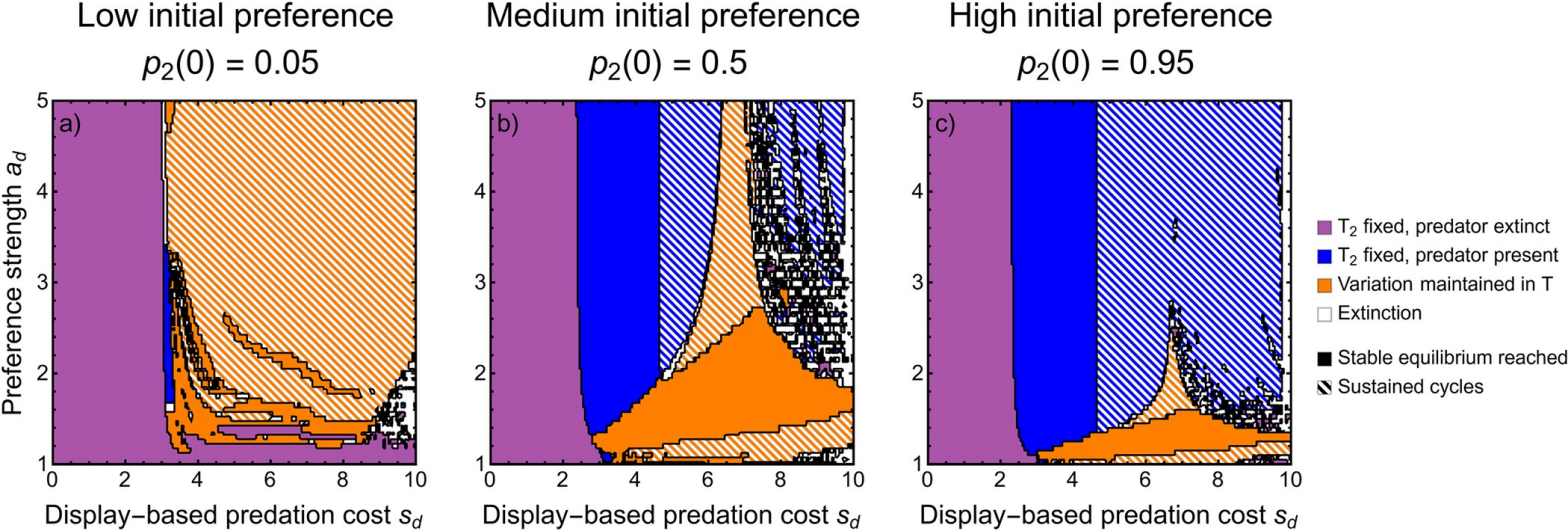
Eco-evolutionary outcomes from the Fisher process model. Parameters correspond to Fig 4C. Note that these are not equilibrium outcomes, but rather the outcome after 2,000 time steps, and thus cycles (striped regions) may not be sustained indefinitely. Apparent black regions are boundaries of two regions that have not been cleanly differentiated in 2,000 time steps. Solid color corresponds to a stable equilibrium being reached and striped corresponds to sustained or long transient cycles. Panels show different values for initial frequency of females with a preference (0.05, 0.5, and 0.95, moving left to right). This Figure can be generated using S1 Code.

Typical explanations for the evolutionary elaboration of female preferences require that they become genetically correlated with the display trait (the Fisher process) [85]. This occurs naturally upon extending the discrete model to consider evolving preferences at an additional locus that controls whether females mate randomly or prefer to mate with displaying males (with preference strength *a*_*d*_; see Methods). Under the Fisher process, numerical iteration of the recursion equations shows that variation in the sexual display is more likely to be maintained (orange) and eco-evolutionary cycles are more frequent (especially with low initial preference frequency; Fig 5A). Note that in this model, cycles may be long transients but not sustained indefinitely (see Methods for details). Preference itself may cycle due to density-dependent predation (Fig 1F), but these cycles have small amplitude since preferences only evolve due to indirect selection. Unsurprisingly, increasing the initial frequency of mating preference in the population expands the parameters for which the male display trait fixes (Fig 5), with results converging to the discrete model without coevolving preferences when the preference frequency is close to 1 (Fig 5C).

## Discussion

Mechanistically modeling the cost of carrying a sexual display as increased predation risk alters both ecological and evolutionary dynamics. The presence of an evolving sexual display reduces the amplitude of ecological cycles by buffering changes in population density (i.e., both density and the trait value can respond). More dramatic changes are seen in evolutionary outcomes. Predation can maintain variation in display traits either through creating a stable interior equilibrium or by driving eco-evolutionary cycles. These changes occur because predation is density dependent, and thus, selection against display traits is also density dependent, since displays increase predation risk.

### Eco-evolutionary dynamics of sexually selected traits

Cycles have previously been seen in sexually selected traits; however, the mechanism behind them is distinct from what we observe here and relies on coevolving female preferences with costs to choosiness. First, cycles can occur when there is an abrupt transition between displays having low costs close to the natural selection optimum to high costs far from the optimum; preferences and displays can then coevolve to extreme values, but costs to choosiness can trigger a slow degradation of preferences and displays promoting rapid evolution to the opposite extreme [86]. Second, cycles can occur in good-genes models when the exhaustion of genetic variation removes the benefit to mate choice, which, in turn, causes the preference to be lost, permitting genetic variation to again build [36]. Evolutionary cycles in our models occur without any of the drivers of cycles in previous models (coevolving female preferences promote cycles in our model but are not necessary; compare Fig 5 to Fig 4).

Rather, cyclic behavior in our models results from fluctuating density-dependent selection that occurs due to predator–prey dynamics. As such, these results relate to the dynamics that occur under the Hamilton–Zuk hypothesis, which posits that sexual displays are often condition dependent and reflective of an individual successfully resisting pathogens [87]. Previous models of that process find that cyclic coadaptation between parasites and hosts may lead to cycles in sexually selected traits [88,89]; however, unlike our model, these studies assume fluctuating selection rather than explicitly modeling host–pathogen dynamics. Given that evolution can dampen ecological cycles (e.g., Figs 1E and S1), past models that have not included explicit eco-evolutionary dynamics may have overestimated ecological cycles and, hence, evolutionary cycles.

Our results closely relate to previous work on eco-evolutionary dynamics in predator–prey systems. Stability in coevolving predator–prey systems has been argued to require a faster life history in predators than prey [90]. The current work supports this result by showing that increasing predator death rate *m* promotes stability (i.e., inhibits cycles; Fig 3). Our results are also consistent with previous work showing that faster prey adaptation is destabilizing [77] (S3 Fig). Moving to characteristics of observed cycles, sexual selection increases the lag from the prey peak to the predator peak (S2E Fig), but cycles are never reversed (i.e., the lag is always less than half the period). Reversed cycles require prey defense against predation to have a strong positive impact on prey growth rate [74]. The evolution of the sexual display relies on increased reproductive success and does not increase growth rate. Therefore, even though the sexual display is ostensibly analogous to reduced prey defense by increasing predation risk, it does not result in reversed cycles, highlighting an important difference between evolution via sexual selection in predator–prey systems and the evolution of prey defense.

### Coevolution with preference increases the likelihood of cycles

We cannot be certain that cycles are sustained indefinitely when we model the Fisher process with coevolving preferences because we must rely on numerical iteration of the recursion equations (unlike in the other models presented; see S1 Text). Nevertheless, the observed cycles are biologically relevant, maintained for at least thousands of time steps. We show that allowing sexual displays to coevolve with female preferences makes eco-evolutionary cycles more frequent. This follows from weak sexual selection (low preference strength) favoring eco-evolutionary cycles in the discrete model (striped orange, Fig 4) and explicitly modeling mating preferences expands the regions of parameter space under which preferences are weak. A second reason that coevolving female preferences could facilitate eco-evolutionary cycles is that it results in fluctuations in the strength of sexual selection as the display cycles. Thus, initial, transient cycles that are often observed may be exacerbated by cycling preferences. This likely contributes relatively little to cycling, however, since the amplitude of cycles in female preferences is small and thus the fluctuation in the strength of sexual selection also small. The effect of cycling female preference could be exaggerated if females suffered from costs to choice paid through additional predation risk from mating with displaying males, as this would cause sexual selection for displays to weaken under the same conditions as when natural selection against displays strengthens. However, for the presence of costs to choice to not lead to the loss of preferences, including in our model [91], additional assumptions are required [92] (e.g., biased mutation on the display [93]).

### Future directions

One notable omission from our model is that predators cannot evolve. Coevolutionary feedbacks between predator offensive ability and prey defense may qualitatively alter eco-evolutionary dynamics [26,74–76,79,83,90,94–98], making this a valuable future extension. Since sexual displays do not provide an ecological benefit to the prey, the coevolution of predator offensive ability may lead to substantial differences from previous models that include predator evolution. One possibility is that the predator’s search image of the prey will coevolve with sexually selected traits. This could be a particularly interesting extension if females also suffer from increased predation risk when mating with displaying males. In such cases, female preferences could switch to avoid the predator’s search image, a result that could account for the lack of sustained mating preferences in nature in some systems [99,100] and could also enhance variation in sexual displays.

Other notable limiting assumptions include fixed genetic variation in the continuous model and that expression of the display is not conditional on predator density. Dynamic genetic variation is, however, addressed in the discrete model. And, if sexual displays were expressed conditional on predator density being sufficiently low, then we could see similar trait–density dynamics without evolutionary change in the displays. However, a recent meta-analysis suggests that conditional expression of displays is rare, but the strength of mate choice being conditioned on predation risk (e.g., in Trinidadian guppies [101]) seems common [102]. Such conditional choosiness would likely exacerbate eco-evolutionary cycles because the benefit of expressing the display would decrease in predator-rich settings.

A final simplifying assumption is the consideration of only a two-species community. Intuitively, one might expect that a larger community with a generalist predator would decouple the predator from prey evolution. However, adding a second prey species to a previous eco-evolutionary model was found to be typically destabilizing [103]. This suggests that it is unlikely that additional prey species would change our primary conclusion—sexual selection in predator–prey systems produces rich eco-evolutionary dynamics—even if the exact nature of the dynamics was altered.

Connecting model outcomes to natural populations will require measuring the dynamics of predator–prey systems and sexual displays through time. Though challenging, recent work measuring how predators shape trait dynamics through time given a growth/predation–risk trade-off [104,105] could serve as a blueprint. The success in characterizing both the influence of and mechanism behind the growth/predation–risk trade-off [106–108] demonstrates the value of empirical studies on traits favored by sexual selection that increase predation risk. In the interim, our model cautions that short-term measurement of sexual display evolution may hide much richer dynamics occurring over a longer timescale.

### Broader conclusions

Our results have a number of broad implications for the study of sexual selection and eco-evolutionary dynamics. First, they provide a concrete example of eco-evolutionary dynamics in the context of sexual selection, supporting calls for more attention to be paid to the intersection of these concepts [59,60]. Second, both the presence of eco-evolutionary cycles (and the maintenance of polymorphism in the discrete model) show that heightened predation risk could explain sustained variation in sexual displays despite persistent female choice (i.e., the lek paradox). As a possible example, parasitoids have been shown to have induced variation in the sexual displays of Pacific field crickets (*Teleogryllus oceanicus*) [109]. Third, and relatedly, cycles in sexual displays have the potential to mislead empirical studies. For example, if a study measured strong net positive selection on a display, researchers could draw conclusions regarding the directional elaboration of the display. However, our results show that this could depend heavily on the current ecological context and that the long-run dynamics may be cyclic and not resemble the extrapolation of the short-term measurement. Thus, evolution (even via sexual selection) must be viewed as a dynamic, context-dependent process [110]. Empirical studies examining whether there are cycles in sexual displays that are known to increase predation risk would thus be a valuable contribution.

Broadly, our results provide a striking example of the importance of mechanistic modeling in ecology and evolution [110,111]. Considerable evidence supports the widespread verbal argument that sexual displays increase predation risk (e.g., by reducing maneuverability or increasing conspicuousness), constraining display evolution [1–17]. The mechanism of predation as the source of natural selection against sexual displays cannot be captured by standard models of sexual selection that assume the selection coefficient is frequency independent and population densities fixed [18,19], because predation is density dependent. We have shown that the density-dependent nature of predation causes previously unappreciated complexity in the eco-evolutionary dynamics of predator–prey systems with sexual selection in the prey.

## Methods

### Continuous model

The continuous model assumes population and trait changes occur in continuous time and that a normally distributed quantitative trait *z* with mean value $\bar{z}$ controls the degree of sexual display in males. Population dynamics follow the Rosenzweig–MacArthur model [112] with logistic prey growth and a type II function response (predation rate saturates with increasing prey density). Consistent with past work [70], a type I (linear) functional response never leads to evolutionary cycles due to prey evolution and do not consider it further.

We assume a male prey individual with trait value *z* suffers from a “basal” predation rate *b*_*c*_ in the absence of sexual displays and that each unit increase in trait values for sexual displays results in an additional rate of predation of *s*_*c*_ (*s*_*c*_ will be referred to as a display-based predation cost). That is, the attack rate between a single male prey individual and single predator individual that is not actively consuming prey is given by *b*_*c*_+*s*_*c*_*z*. Female prey individuals do not express the display and thus are only attacked at rate *b*_*c*_. Note that to be precise, we use subscript *c* for all continuous model parameters that have an analog in the discrete model. Analogous to Abrams and Matsuda [70], we assume that the predation rate saturates with respect to the overall attack rate (rather than just prey density), since this ultimately determines how often prey are captured. Let *N*_*f*_ be the density of female prey, *N*_*m*_ the density of male prey, and *P* the density of predators. Integrating over the entire prey population, and making the standard mass action assumptions for predator and prey encounters, the total predation rate is given by $\frac{(b_{c}+s_{c}\bar{z})N_{m}P}{1+t_{h}(b_{c}(N_{f}+N_{m})+s_{c}\bar{z}N_{m})}$ in males and $\frac{b_{c}N_{f}P}{1+t_{h}(b_{c}(N_{f}+N_{m})+s_{c}\bar{z}N_{m})}$ in females. Here, *t*_*h*_ denotes the handling time (the duration of consumption) [113]. Increasing handing time corresponds to faster saturation of the predation rate. With these assumptions, the dynamics of prey density follow

$$\frac{dN_{f}}{dt}=r_{c}N_{f}(1-N_{f}-N_{m})-\frac{b_{c}N_{f}P}{1+t_{h}(b_{c}(N_{f}+N_{m})+s_{c}\bar{z}N_{m})}$$ (1A)

$$\frac{dN_{m}}{dt}=r_{c}N_{f}(1-N_{f}-N_{m})-\frac{(b_{c}+s_{c}\bar{z})N_{m}P}{1+t_{h}(b_{c}(N_{f}+N_{m})+s_{c}\bar{z}N_{m})}.$$ (1B)

The first term reflects that the prey population grows logistically in the absence of predation with intrinsic rate of increase *r*_*c*_ and carrying capacity set arbitrarily to one. Note that births depend only on the presence of females in the population, but competition occurs equally with both males and females. The second term models predation.

We assume that predators convert prey biomass with efficiency *c*_*c*_ and experience a constant per capita mortality rate *m*. Thus, predator dynamics obey

$$\frac{dP}{dt}=c_{c}\frac{\left(b_{c}(N_{f}+N_{m})+s_{c}N_{m}\right)}{1+t_{h}(b_{c}(N_{f}+N_{m})+s_{c}\bar{z}N_{m})}-mP.$$ (2)

The first term accounts for predators converting each unit of prey biomass that they capture into *c*_*c*_ units of predator biomass, and the second term accounts for predator mortality.

Now that ecological dynamics have been fully specified, we turn to deriving evolutionary dynamics. Following Lande [18], we partition the selection gradient into its natural and sexual selection components. The natural selection component is given by $\frac{\partial }{\partial z}\frac{1}{N_{m}}\frac{dN_{m}}{dt}=-\frac{s_{c}P}{1+t_{h}(b_{c}(N_{f}+N_{m})+s_{c}\bar{z}N_{m})}$, where we interpret *z* in the numerator of Eq (1B) as an individual’s trait and $\bar{z}$ in the denominator as the population average. Note that natural selection disfavors displays proportional to predator density. The sexual selection gradient is assumed to follow Lande’s “absolute” preference function [18], such that females prefer males with trait value *y* and the strength of their preference is *a*_*c*_. We arbitrarily set *y* = 1 such that the sexual selection component of the selection gradient is $\frac{1-\bar{z}}{\frac{1}{a_{c}}+V(\bar{z})}$, where *V* is the additive genetic variation. Following Abrams and Matsuda [70], we choose *V* to be nearly constant except as the display approaches its minimum value of 0 to bound the trait from below. In particular, we use $V(\bar{z})=\sigma^{2}Exp[\frac{-\epsilon }{\epsilon +\bar{z}}]$. Here, *σ*^2^ is the amount of heritable phenotypic variation when $\bar{z}\gg \epsilon$ and *ϵ* is a small positive number that controls that scale at which variation goes to 0 as $\bar{z}$ goes to 0. We set *ϵ* = 10^−3^. We use the gradient dynamics approach [114], wherein the trait under selection evolves proportional to its selection gradient, to model evolutionary dynamics. Though often assumed, gradient dynamics has been formally justified for cases when the third and higher odd order derivatives of fitness with respect to the evolving trait are small or vanish. Here, setting the genetic variance constant, which is approximately true unless the display becomes quite weak, we see that the third derivative of our fitness function is $\frac{\partial^{3}}{\partial z^{3}}\left[-\frac{s_{c}zP}{1+t_{h}(b_{c}(N_{f}+N_{m})+s_{c}\bar{z}N_{m})}+\int \frac{1-z}{\frac{1}{a_{c}}+\frac{\sigma^{2}}{2}}dz\right]=0$, and thus, gradient dynamics can be used for our system. The dynamics of the evolving display then follow

$$\frac{d\bar{z}}{dt}=V(\bar{z})\left(\frac{1-\bar{z}}{\frac{1}{a_{c}}+V(\bar{z})}-\frac{s_{c}P}{1+t_{h}(b_{c}(N_{f}+N_{m})+s_{c}\bar{z}N_{m})}\right).$$ (3)

The first term in the parentheses is selection resulting from mate choice (positive so long as $\bar{z}<1$: females prefer more extravagant sexual displays), and the second term in the parentheses is selection resulting from predation (negative because predators are more likely to capture prey that are expressing a more extreme sexual display). Eqs (1)–(3) make up the continuous model.

### Discrete model

The discrete model assumes nonoverlapping generations evolving in discrete time with a single haploid locus T that controls the expression of a sexual display in males. Males with the T_2_ allele express the display, whereas males with the T_1_ allele do not. Now that the sexual display is a sexually dimorphic discrete trait, we can write *N = N*_1,*F*_ + *N*_1,*M*_ + *N*_2,*F*_ + *N*_2,*M*_, where *N*_1,*F*_ is the density of females carrying the T_1_ allele, *N*_1,M_ is the density of males carrying the T_1_ allele (nondisplaying males), *N*_2,*F*_ is the density of females carrying the T_2_ allele, and *N*_2,*M*_ is the density of males carrying the T_2_ allele (displaying males). It is also convenient to define *N*_*i*_ = *N*_*i*,*F*_ + *N*_*i*,*M*_ as the density of individuals with the T_*i*_ allele.

The first step is predation. Again, it is assumed that *b*_*d*_ is a basal predation rate and that *s*_*d*_ is the additional predation cost resulting from expressing a sexual display. Note the subscript *d* making explicit that these are parameters of the discrete model. The densities of *N*_1_ and *N*_2_ individuals of each sex following predation (denoted with a prime) are

$$N_{1,F}^{\prime }=N_{1,M}^{\prime }=(1-b_{d}P(t))\frac{N_{1}(t)}{2}$$ (4A)

$$N_{2,F}^{\prime }=(1-b_{d}P(t))\frac{N_{2}(t)}{2}$$ (4B)

$$N_{2,M}^{\prime }=(1-(b_{d}+s_{d})P(t))\frac{N_{2}(t)}{2},$$ (4C)

where dividing by two in the final term accounts for the assumption of equal sex ratio at birth. Notice that only males carrying the T_2_ allele suffer from additional mortality. Whenever (*b*_*d*_ + *s*_*d*_) *P* > 1, the model is no longer well behaved and prey density becomes negative, which we define to be extinction. We always check for this possibility in presenting results other than stable equilibria: It only occurs when *s*_*d*_ or *b*_*d*_ are quite high.

Following mortality, the surviving prey mate, following the assumptions of Kirkpatrick [19]. Prey are assumed to be polygynous (every female has equal mating success). Females are *a*_*d*_ times more likely to mate with a displaying (T_2_) male upon encounter (*a*_*d*_ will again be referred to as preference strength). Under these assumptions, the number of females with the *j* allele paired with a male with the *i* allele is

$$M_{ij}=\frac{\chi_{i}N_{i,M}^{\prime }N_{j,F}^{\prime }}{N_{1,M}^{\prime }+a_{d}N_{2,M}^{\prime }},$$ (5)

where *χ*_*i*_ is an indicator function accounting for female preference and evaluates to 1 if *i* = 1 and evaluates to *a*_*d*_ if *i* = 2. The denominator of Eq (5) normalizes the mated pairs such that all females will have equal mating success. The frequency of genotypes in the next time step follow from the standard assumptions of haploid genetics. Namely,

$$t_{2}=\frac{M_{22}+\frac{1}{2}(M_{12}+M_{21})}{\sum_{i,j}M_{ij}}$$ (6)

is the frequency of the T_2_ allele at the beginning of time step *t* + 1.

All that remains is to determine the density of the predator and prey populations at the next time step. We assume that density-dependent prey growth follows the logistic map such that

$$N(t+1)=r_{d}N_{f}^{\prime }(1-N^{\prime }),$$ (7)

where *N*′ is the total density of prey following predation and $N_{f}^{\prime }$ is the density of females after predation. As with the continuous model, this assumes that the number of births is limited only by females but that all prey individuals compete equally. Predators are also assumed to reproduce with nonoverlapping generations. Thus, the predator density at the beginning of time *t* + 1 is

$$P(t+1)=c_{d}P(t)(b_{d}(N_{1}(t)+N_{2}(t))+\frac{s_{d}}{2}N_{2}(t)),$$ (8)

where *c*_*d*_ is the conversion efficiency and *P*(*t*) times the term in the parentheses is the total amount of prey biomass consumed in time step *t*.

### Model analysis

We determined equilibrium outcomes using linear stability analysis. Equilibria and Jacobian matrices were determined analytically. In the continuous model, we set $V(\bar{z})=\sigma^{2}$ for analytical tractability (see S1 Text). Cases with no stable equilibria were analyzed numerically to determine (1) if the system remained well behaved, (2) what type of cycles occur, and (3) quantitative features of the cycles using the EcoEvo package [115]. Details about analyses can be found in the S1 Text.

### Fisher process model

A major problem in sexual selection theory is understanding not just the evolution of display traits but also the evolution of preferences for the displays. One widespread explanation for the evolution of female preferences is the Fisher process [85]: Alleles encoding preference become correlated with display traits and thus increase in frequency due to indirect selection. Our discrete model is closely related to a model of the Fisher process [19]. To assess whether density-dependent selection against display traits has important implications for the evolution of female preference we extended the discrete model to include the Fisher process (though, of course, one could also develop a continuous model of the Fisher process [18]).

The model follows closely from Kirkpatrick [19], with previously described alterations such that predation risk mechanistically accounts for the cost to expressing a sexual display. Now, there are two loci: In addition to the T locus that controls the display, the P locus controls female preference. Females carrying the P_1_ allele mate randomly, whereas females carrying the P_2_ allele prefer to mate with males that express the sexual display (with preference strength *a*_*d*_) as before. We assume that an individual’s allele at the P locus does not influence their predation risk and that all females have equal mating success regardless of the allele they carry at the P locus (strict polygyny). Thus, our assumptions governing mortality remain unchanged and our mating equations follow from Kirkpatrick [19] (full equations can be found in the S1 Code). We assume free recombination between the T and P loci. Our Fisher process model is not analytically tractable, and thus, all analyses were carried out through numerical iteration of the recursion equations (see S1 Text).

### Assessing the role of eco-evolutionary dynamics

The primary claim of our study is that explicitly modeling the cost of sexual displays as increasing predation risk leads to qualitatively different outcomes than is seen in comparable systems without sexual selection or without predator–prey dynamics. To drive home the importance of eco-evolutionary feedbacks in our model, we “turn off” ecology and evolution, sequentially, in both of our models to determine how outcomes are altered.

We begin with the continuous model. The evolution-only continuous model is straightforward to create. One must only fix *P* in the equation for $\frac{d\bar{z}}{dt}$ to remove ecological dynamics from selection. We begin by fixing *P* = 0.1, arbitrarily. The ecology-only continuous model is also simple to create. In this case, it is necessary to fix $\bar{z}$ in the equations for $\frac{dN_{i}}{dt}$ and $\frac{dP}{dt}$ to remove evo-to-eco feedbacks. Specifically, we fix $\bar{z}=1$. Results from this analysis can be found in S1A–S1C Fig.

The procedure described in the previous paragraph may result in model outcomes that are different than the eco-evolutionary model for two reasons. First, the absence of eco-evolutionary feedbacks per se may change results. Second, different long-run values of *P* and $\bar{z}$ may change results. To tease apart these two possibilities, we first fix *P* in the evolution-only model so as to make the results as comparable as possible to the full, eco-evolutionary model. If the predator density approaches a stable equilibrium in the full, eco-evolutionary model, then we use the stable value for predator density as *P* in the evolution-only model. If sustained cycles occur, then we use the average predator density over the course of its cycle as *P* in the evolution-only model. Analogous to the evolution-only model, we also chose the fixed value of $\bar{z}$ to make the results from the ecology-only model as comparable as possible to the full, eco-evolutionary model. Specifically, when $\bar{z}$ approaches a stable equilibrium in the eco-evolutionary model, we fix the stable equilibrium value of $\bar{z}$ in the ecology-only model. When sustained cycles occur, we use the mean display value over the course of the cycle as $\bar{z}$ in the ecology-only model. Results from this analysis can be found in Fig 2A–2C.

We now turn to the discrete model, using the same two analyses as above. First, we simply fix the display as present in the discrete model when forming the ecology-only discrete model and fix the predator density at an arbitrary value of *P* = 0.03 when forming the evolution-only discrete model (shown in S1D–S1F Fig). Second, we again use the stable equilibria or mean value in a cycle to make the models more comparable. In this case, we dealt with polymorphism by setting the death probability of males as $b_{d}+t_{2}^{*}s_{d}$ where $t_{2}^{*}$ is the stable (or mean) frequency of the display from the eco-evolutionary model. This analysis is shown in Fig 2D–2f.

## Supporting information

S1 Fig The role of predator–prey dynamics and sexual selection in shaping model outcomes. (PDF)

S2 Fig Correlation between cycle measurements in the continuous model. (PDF)

S3 Fig The effect of additional parameters in the continuous model. (PDF)

S4 Fig Polymorphism and cycles in the discrete model. (PDF)

S1 Text Supplementary methods. (PDF)

S1 Code Mathematica notebook to replicate results. (NB)
