# Correction: Predation drives complex eco-evolutionary dynamics in sexually selected traits

**DOI:** 10.1371/journal.pbio.3003125

**Published:** 2025-04-04

**Authors:** Brian A. Lerch, Maria R. Servedio

In the Continuous model subsection of the Methods, there is a typo in the second equation. The numerator is incorrect. This error was not present in the code used for analyses and thus the results are unaffected. Please view the complete, correct equation here:


$$\frac{dP}{dt}=c_{c}\frac{P(b_{c}\left(N_{f}+N_{m}\right)+s_{c}\bar{z}N_{m})}{1+t_{h}\left(b_{c}\left(N_{f}+N_{m}\right)+s_{c}\bar{z}N_{m}\right)}-mP$$
