# Supplementary material for: Predation drives complex eco-evolutionary dynamics in sexually selected traits
Source: PLoS Biol. 2023 Apr 3;21(4):e3002059. doi: 10.1371/journal.pbio.3002059 (PMC10101644; doi:10.1371/journal.pbio.3002059)
Supplement: S1 Fig — (PDF) [file pbio.3002059.s001.pdf]

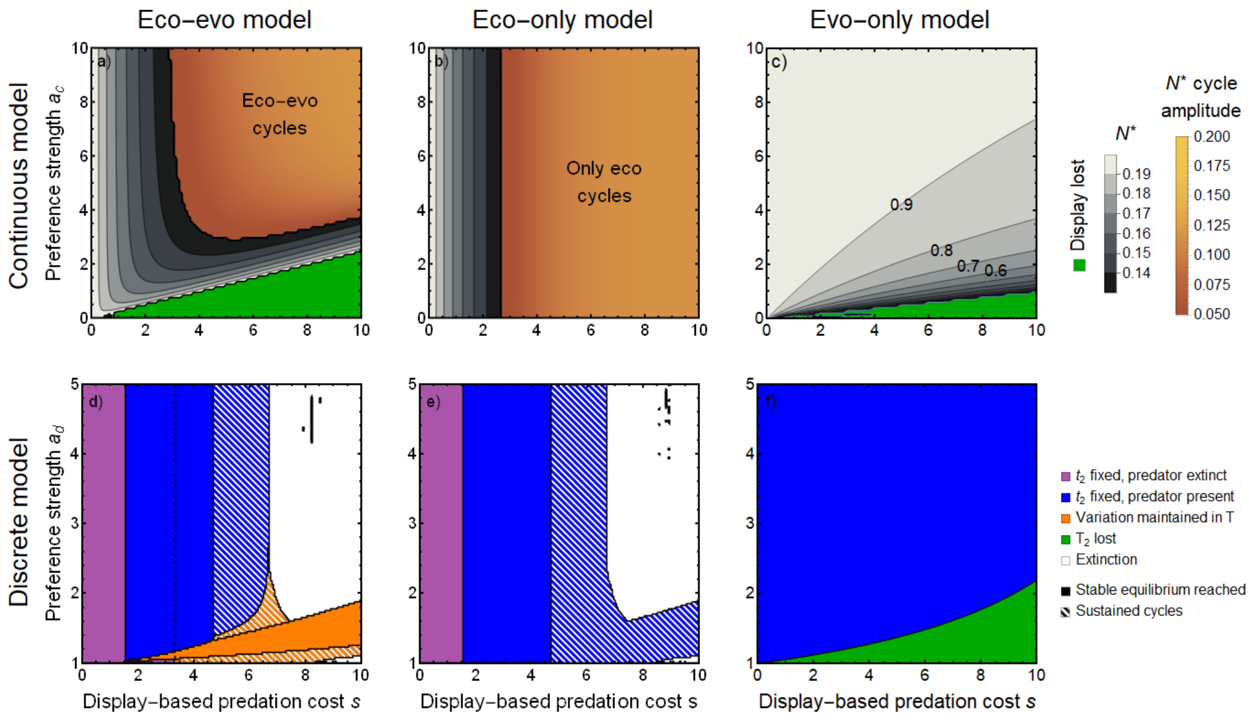

S1 Fig. The role of predator-prey dynamics and sexual selection in shaping model outcomes with arbitrary, fixed trait values and densities (see Methods). This provides the same information as Fig. 2 (with the same parameters), but does not use trait values, frequencies, and densities from the full eco-evolutionary model when generating the ecology- and evolution-only models. The first row shows the continuous model. (a) Full eco-evolutionary model. (b) Ecology-only model with $\bar{z} = 1$. (c) Evolution-only model with $P = 0.1$. The second row shows the discrete model. (a) Full eco-evolutionary model. (b) Ecology-only model with $t_2 = 1$. (c) Evolution-only model with $P = 0.03$. This Figure can be generated using S1 Code.
