# Supplementary material for: Predation drives complex eco-evolutionary dynamics in sexually selected traits
Source: PLoS Biol. 2023 Apr 3;21(4):e3002059. doi: 10.1371/journal.pbio.3002059 (PMC10101644; doi:10.1371/journal.pbio.3002059)
Supplement: S2 Fig — (PDF) [file pbio.3002059.s002.pdf]

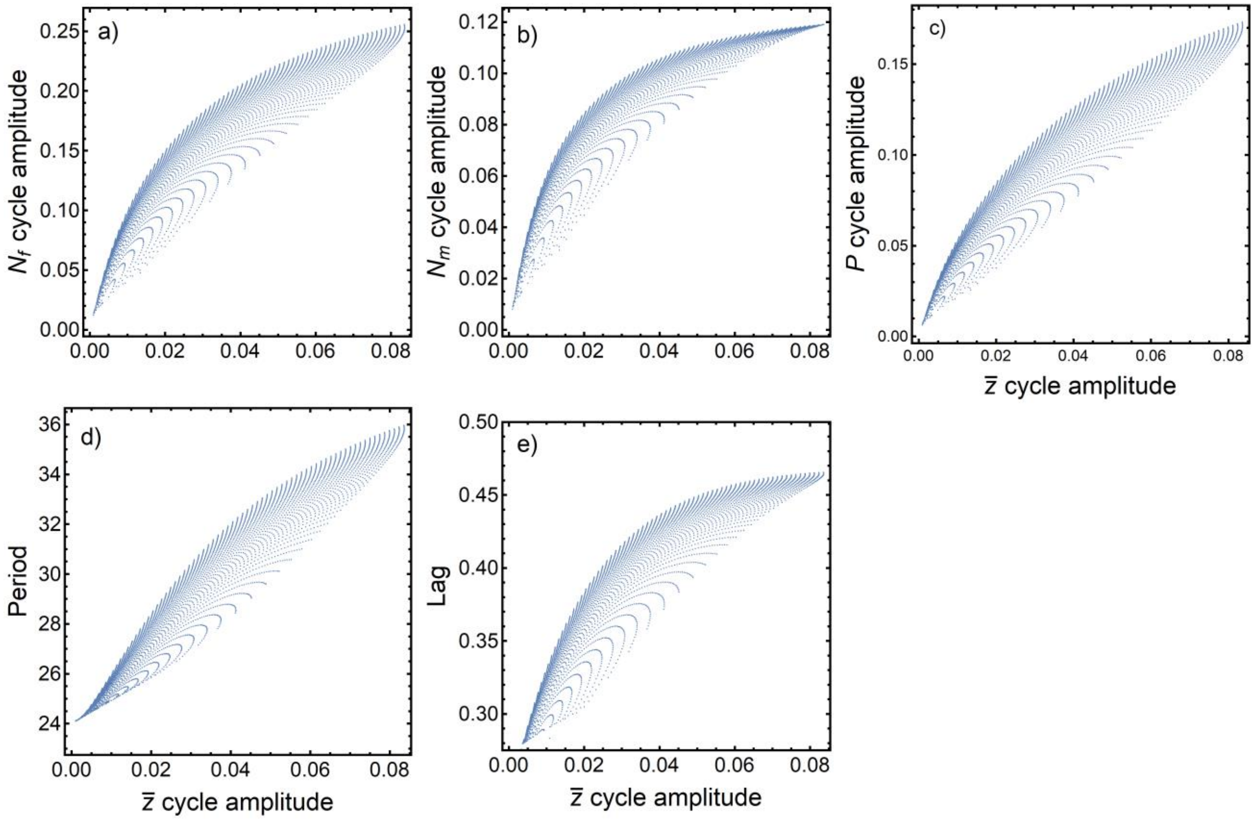

S2 Fig. Correlation between various cycle measures in the continuous model with each point representing a single simulation from the region with eco-evolutionary cycles in Fig 3b. High amplitude cycles in the display imply high amplitude cycles in (a,b) prey and (c) predator densities. (d) Higher amplitude cycles have longer period. (e) The predator peak occurs later in the prey cycle as the amplitude of the display cycle increases, and almost always occurs between quarter- and half-way through the prey cycle. This Figure can be generated using S1 Code.
