# Supplementary material for: Predation drives complex eco-evolutionary dynamics in sexually selected traits
Source: PLoS Biol. 2023 Apr 3;21(4):e3002059. doi: 10.1371/journal.pbio.3002059 (PMC10101644; doi:10.1371/journal.pbio.3002059)
Supplement: S3 Fig — (PDF) [file pbio.3002059.s003.pdf]

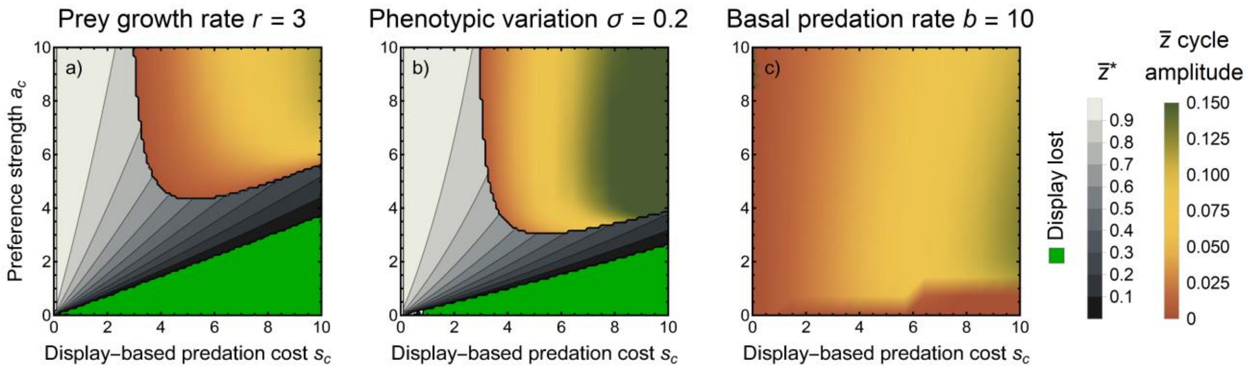

S3 Fig. Effect of additional parameters in the continuous model (each panel uses the same parameters as Fig 3b, except for the change noted above the panel). (a) Increasing prey growth rate r_c slightly decreases the parameter range over which eco-evolutionary cycles occur, slightly increase the range over which the display is lost, and increases the amplitude of cycles. (b) Increasing prey genetic variation σ increases the amplitude of cycles but otherwise has little effect. (c) Increasing the basal predation rate b_c greatly increases the range of parameters under which eco-evolutionary cycles occur. Note that the display is lost in the rust-colored region in the bottom right and thus these are only ecological cycles. This Figure can be generated using S1 Code.
