# Supplementary material for: Predation drives complex eco-evolutionary dynamics in sexually selected traits
Source: PLoS Biol. 2023 Apr 3;21(4):e3002059. doi: 10.1371/journal.pbio.3002059 (PMC10101644; doi:10.1371/journal.pbio.3002059)
Supplement: S4 Fig — (PDF) [file pbio.3002059.s004.pdf]

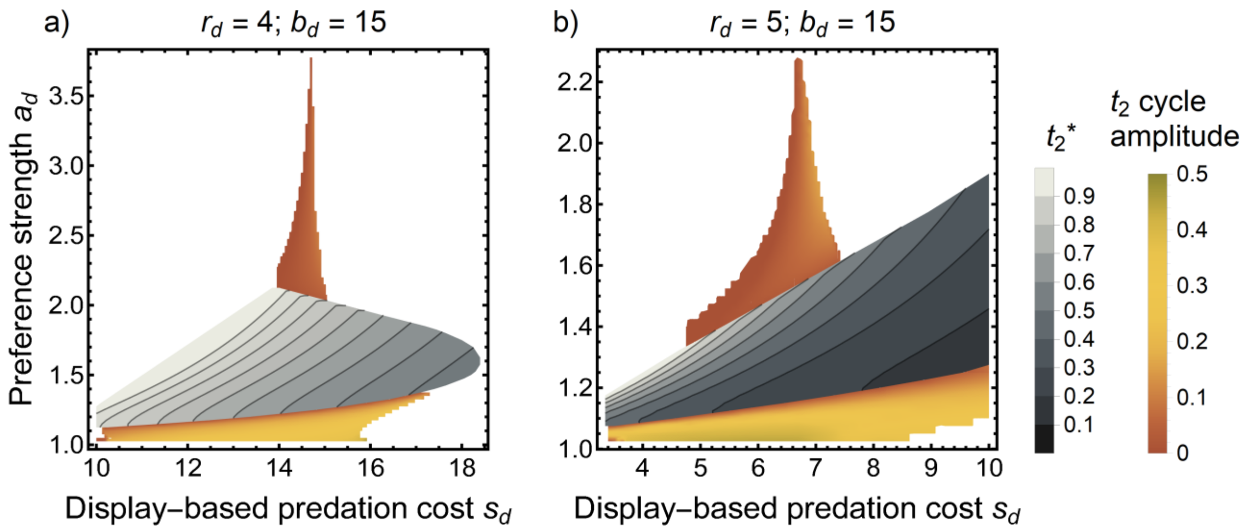

S4 Fig. Degree of polymorphism maintained and amplitude of cycles in the discrete model. Gray-scale region indicates a stable polymorphism, with lighter colors corresponding to higher frequency of the display at equilibrium. Yellow-to-red regions indicate sustained eco-evolutionary cycles, with yellow-green colors corresponding to higher amplitude cycles. c_d = 0.1. This Figure can be generated using S1 Code.
