# Supplementary material for: Predation drives complex eco-evolutionary dynamics in sexually selected traits
Source: PLoS Biol. 2023 Apr 3;21(4):e3002059. doi: 10.1371/journal.pbio.3002059 (PMC10101644; doi:10.1371/journal.pbio.3002059)
Supplement: S1 Text — (PDF) [file pbio.3002059.s005.pdf]

S1 Text: Supplementary Methods

Model Analysis

The equilibria of both models were determined analytically as were the systems' Jacobian matrices. To determine model outcomes at a particular set of parameter values, we first checked for equilibria that were biologically feasible (non-negative and real). We then performed a linear stability analysis for all biologically feasible equilibria. If there was a single stable, biologically feasible equilibrium, then we characterized the model outcome based on the qualitative features of the equilibrium (i.e., prey extinct, sexual display fixed, etc.). If there were no stable, biologically feasible equilibria for a particular set of parameters we numerically integrated (or iterated, for the discrete model) the model for 1000 time units to 1) ensure trajectories remained well behaved and 2) characterize the qualitative features of trajectories (i.e., prey extinct, sexual display fixed, cycle measurements, etc.).

For the continuous model, slight modifications were needed for analytical tractability. Namely, we set $V(\bar{z}) = \sigma^2$ for analytical calculations (though always included the more complex dependence on trait value for numerical integration). Since our more complex function for $V(\bar{z}) \approx \sigma^2$ unless $\bar{z}$ approaches 0, equilibrium outcomes can safely be extrapolated from this simpler model, with one exception: the loss of the sexual display. We impose a lower bound on $\bar{z}$ of 0 by assuming a loss of genetic variation in this limit, though the selection gradient does not necessarily approach 0 as $\bar{z}$ approaches 0. To determine whether the display is lost from the continuous model, we set $\bar{z} = 0$ and analyze the resulting model of only predator-prey dynamics without evolution. In this situation, there is only one equilibrium for which the predator may persist: $\left(N_f^*, N_m^*, P^*\right) = \left(\frac{m}{2b_c(c_c - mt_h)}, \frac{m}{2b_c(c_c - mt_h)}, \frac{c_c r_c(b_c(c_c - mt_h) - m)}{\left(b(c - mt_h)\right)^2}\right)$. If (1) this equilibrium is

stable in the three-dimensional system and (2) the selection gradient is negative when the predator density is equal to P^*, then the display is lost and the eco-evolutionary dynamics can approach a stable equilibrium of $\left(N_f^*, N_m^*, P^*, z^*\right) =$ $\left(\frac{m}{2b_c(c_c-mt_h)}, \frac{m}{2b_c(c_c-mt_h)}, \frac{c_c r_c(b_c(c_c-mt_h)-m)}{\left(b(c-mt_h)\right)^2}, 0\right)$.

We rely on the numerical procedure described above because the full generality of the equilibria and their stability conditions are too complex to be meaningfully interpreted. Still, some special cases are illuminating. In addition to cases where the display is lost in the continuous model, we identified six equilibria in the continuous model (though not all of these will be biologically feasible for specific parameter values). Two of these equilibria are complex and include both non-zero predator and prey densities and non-trivial (not 0 or 1) trait values. The remaining four include extinction of the predator. One of these, $\left(N_f^*, N_m^*, P^*, \bar{z}^*\right) =$ $(0,0,0,1)$, also includes extinction of the prey, and is always unstable. Another equilibrium includes no females but the presence of males and is thus always infeasible. The last two equilibria are potentially feasible with the display at its sexually selected optimum and the prey at non-zero densities. In practice, we assume an equal sex ratio in the absence of predation and check if the per capita predator growth rate is positive to assess the stability of predator extinction. In particular, this means that predator extinction is stable when $m > \frac{c(b_c+s_c/2)}{1+b_c t_h+s_c t_h/2}$, which is simply that the predator mortality rate is greater than the rate that prey biomass is converted into predator biomass.

In the discrete model, we identified nine equilibria (though not all of these will be biologically feasible for specific parameter values). Three of the equilibria include the predator at non-zero densities and polymorphism in the display trait, two include the predator at non-zero

densities and the display at fixation, and two include the predator at non-zero densities and the display trait having been lost from the population. Only the remaining two equilibria with the predator extinct can be analyzed simply. They are loss and fixation of the display trait with prey density at carrying capacity: $(N_1^*, N_2^*, P^*) = \left(\frac{r_d-2}{r_d}, 0,0\right)$ and $(N_1^*, N_2^*, P^*) = \left(0, \frac{r_d-2}{r_d}, 0\right)$. The first equilibrium (loss of the display trait) is always unstable. The second equilibrium (fixation of the display trait) is stable whenever $2 < r_d < 6$ (as is required for stable prey growth) and the eigenvalue $\frac{c_d(r_d-2)(b_d+s_d/2)}{r_d}$ (which is simply the per capita predator growth rate) is less than 1 in magnitude. Bistability is sometimes observed in the discrete model. In practice, we found that this was only the case with the predator-extinction equilibrium. We always report an equilibrium with non-zero predator densities when this is also stable (see Fig 4).

Fisher process model analysis

Although a straightforward extension of our discrete model, the Fisher process model includes an additional two state variables that greatly diminish the analytical tractability of the model. In the Fisher process model, it is not possible (even numerically) to solve for all equilibria. As such, we focus on interesting cases from the discrete model to assess how including the coevolution of female preference alters our results. For each parameter combination we considered, we use initial conditions of 0.5 for the initial frequency of T_2 and assume that the T and P loci are initially in linkage equilibrium. We iterated the model for 2000 time steps for initial P_2 frequencies of 0.05, 0.5, and 0.95. We assessed the qualitative features of trajectories and final time points to characterize model outcomes. Specifically, we checked whether the trajectory remained well behaved, the predator had gone extinct (using a threshold of 10^{-10}), and the display trait had gone to fixation (using a threshold of 10^{-10} for the density of prey

carrying the T_1 allele). For all well-behaved trajectories, we further checked whether the trajectories displayed either ecological (just predator/prey density) or evolutionary (allele frequency) cycles in the final 500 time steps. We considered a variable to be cycling if 1) it changed directions at least twice in the final 500 time steps and 2) the range of oscillations was at least 0.01. We note that this procedure will undoubtedly mark regions as "cycling" that are experiencing damped oscillations toward a stable equilibrium. Thus, we will interpret these regions as long transient cycles that may not be sustained.

References

1. Rosenzweig, M. L. & MacArthur, R. H. Graphical Representation and Stability Conditions of Predator-Prey Interactions. *The American Naturalist* **97**, 209–223 (1963).
2. Abrams, P. A. & Matsuda, H. PREY ADAPTATION AS A CAUSE OF PREDATOR-PREY CYCLES. *Evolution* **51**, 1742–1750 (1997).
3. Holling, C. S. Some Characteristics of Simple Types of Predation and Parasitism. *Can Entomol* **91**, 385–398 (1959).
4. Abrams, P. A., Matsuda, H. & Harada, Y. Evolutionarily unstable fitness maxima and stable fitness minima of continuous traits. *Evol Ecol* **7**, 465–487 (1993).
5. Lande, R. Models of speciation by sexual selection on polygenic traits. *Proceedings of the National Academy of Sciences* **78**, 3721–3725 (1981).
6. Kirkpatrick, M. Sexual Selection and the Evolution of Female Choice. *Evolution* **36**, 1 (1982).
7. Fisher, R. A. *The Genetical Theory of Natural Selection*. (Clarendon Press, 1930).
